# Research advances of tubeless thoracic surgery for pulmonary nodules: current status and future challenges

**DOI:** 10.3389/fsurg.2026.1834893

**Published:** 2026-05-25

**Authors:** Hengyu Li, Lei Zhao, Hui Tian

**Affiliations:** Department of Thoracic Surgery, Shandong Key Laboratory of Digital Diagnosis and Treatment of Thoracic Oncology, Shandong Engineering Research Center of Intelligent Surgery, The First Affiliated Hospital of Shandong First Medical University & Shandong Provincial Qianfoshan Hospital, Jinan, China

**Keywords:** enhanced recovery after surgery (ERAS), paravertebral block, pulmonary nodules, Tubeless technology, video-assisted thoracoscopic surgery (VATS)

## Abstract

**Objective:**

This narrative review aims to summarize the current research progress on Tubeless thoracic surgery for pulmonary nodules, analyze its physiological basis and clinical applications, and outline future directions.

**Methods:**

A literature search was performed in PubMed and Web of Science from database inception to December 2025 using the following Boolean search string: (“Tubeless” OR “non-intubated” OR “spontaneous ventilation”) AND (“VATS” OR “video-assisted thoracoscopic surgery”) AND (“pulmonary nodules” OR “lung nodules” OR “GGN”). A total of 1,902 records were identified. After title and abstract screening independently by two authors, 59 articles were finally included. Eligible studies included randomized controlled trials, prospective and retrospective cohort studies, case series (*n* ≥ 10), and expert consensus statements. Only English-language articles were included, as the review is intended for an international readership. Given the heterogeneous nature of the available evidence, this review is presented as a narrative synthesis with no formal quality assessment or meta-analysis.

**Key findings:**

Data from heterogeneous studies suggest reductions in laryngeal edema, postoperative nausea and vomiting, pain, and hospital stay, although direct comparisons should be interpreted with caution. However, risks include intraoperative hypercapnia and the potential need for conversion to intubation. Long-term oncological safety remains unconfirmed.

**Conclusion:**

In carefully selected patients and experienced centers, available evidence suggests that Tubeless technology may optimize perioperative management and enhance recovery after pulmonary nodule resection. Future efforts should focus on integrating electromagnetic navigation bronchoscopy, artificial intelligence for risk prediction, and day-surgery pathways to maximize clinical and economic benefits.

## 1 Introduction

### 1.1 Clinical challenges of pulmonary nodules and the need for minimally invasive surgery

Lung cancer is the leading cause of cancer-related deaths worldwide, with its high mortality rate primarily attributable to late-stage diagnosis. With the widespread adoption of chest CT in lung cancer screening and health examinations for high-risk populations, the detection rate of pulmonary nodules has experienced unprecedented growth. Epidemiological data indicate that in large-scale screening trials, up to half of participants are found to have at least one pulmonary nodule, with approximately 2%–4% ultimately diagnosed with early-stage lung cancer (1–3). This public health initiative has increased early diagnosis rates, creating opportunities for curative treatment.

The etiology of pulmonary nodules is complex, encompassing a spectrum ranging from benign granulomas and post-infectious scars to malignant primary lung cancers or metastatic tumors. This heterogeneous etiology makes decision-making particularly difficult. For highly suspicious nodules, histological diagnosis remains the established technique. Nevertheless, conventional diagnostic approaches are each limited: percutaneous needle biopsy carries the risk of pneumothorax and hemorrhage; for small peripheral nodules, conventional bronchoscopy achieves a diagnostic yield of only approximately 37%, though this rate can vary depending on lesion characteristics and the use of advanced navigation techniques (4). Once malignancy is confirmed or strongly suspected, surgical resection offers dual value in both diagnosis and treatment. Traditionally, open thoracotomy with lobectomy and systematic lymph node dissection has been the standard approach. However, despite its proven efficacy, the muscle transection and rib retraction required for open surgery result in significant trauma, severe postoperative pain, and prolonged recovery periods, substantially impacting patients' quality of life (5). Consequently, the exploration of safer, minimally invasive diagnostic and therapeutic strategies represents a pressing clinical need. The rationale for Tubeless surgery is most directly applicable to patients with peripheral small nodules (≤2–3 cm), including ground-glass nodules (GGNs), subsolid nodules, and small solid nodules, for whom wedge resection or segmentectomy is planned. For patients requiring lobectomy with systematic lymph node dissection, the evidence for Tubeless approaches remains limited.

### 1.2 The rationale and development of tubeless technology

Currently, video-assisted thoracoscopic surgery (VATS) is the mainstream minimally invasive technique for treating pulmonary nodules. Compared to traditional open surgery, its minimally invasive nature has reduced surgical trauma. However, this technique still relies on endotracheal intubation under general anesthesia and the placement of postoperative chest drainage tubes. While these necessary procedures ensure surgical safety, they also introduce numerous complications, such as: intubation-related throat pain, laryngeal edema, airway injury, drainage tube-related pain, infection, and restricted mobility (6). Despite continual progress in minimally invasive techniques, existing solutions remain inadequate to address the growing clinical demand for enhanced recovery and reduced iatrogenic injury. To minimize these catheter-related complications and further enhance the patient experience, the field of thoracic surgery has driven ongoing technological innovation, leading to the development of Tubeless technology. These technical advantages are particularly relevant to patients undergoing limited resections (wedge or segmentectomy) for peripheral small nodules, where the avoidance of invasive lines has the greatest impact on recovery. The core concept of this approach is to eliminate traditional tracheal tubes, chest drains, and even urinary catheters (7), encompassing several distinct strategies including non-intubated anesthesia, omission of chest drainage, and avoidance of urinary catheters, while employing an anesthetic technique that preserves spontaneous ventilation. These strategies are not always used together in clinical practice. Some studies omit only the chest tube under intubated anesthesia, while others focus solely on non-intubated anesthesia with a chest tube still in place. For clarity, this review uses the term “Tubeless” to refer to the combination of all three components unless otherwise specified. See Table 1 for definitions of these components. Its primary advantages are manifested in: The preservation of spontaneous breathing maximally protects pulmonary function during surgery, reducing mechanical lung injury. Avoiding tube placement directly alleviates postoperative pain (with significantly lower postoperative VAS scores) and promotes early mobilisation and recovery (11). Currently, Tubeless technology has evolved from conceptual exploration to become a feasible and safe option for selected patients with pulmonary nodules. However, the successful implementation of Tubeless techniques requires careful patient selection and is associated with a learning curve, and its widespread adoption remains limited by concerns over intraoperative emergencies and the lack of long-term oncological outcome data. In summary, Tubeless technology not only complements and advances VATS techniques but also represents a significant advancement in the minimally invasive philosophy of thoracic surgery and the practice of Enhanced Recovery After Surgery (ERAS). This review systematically elaborates on the theoretical foundations, clinical applications, and future challenges of Tubeless technology, aiming to offer new perspectives for optimising diagnostic and therapeutic strategies for pulmonary nodules.

**Table 1:** Definitions of key components in Tubeless strategies.

Term	Definition
Non-intubated anesthesia	No endotracheal tube; LMA or spontaneous breathing without positive-pressure ventilation
Spontaneous-ventilation VATS	Patient maintains spontaneous breathing throughout the procedure
Awake surgery	Patient remains conscious or under minimal sedation; no artificial airway
Chest-drain omission	No chest tube placed after surgery
Urinary-catheter avoidance	No urinary catheter placed
Fully Tubeless pathway	Combination of non-intubated anesthesia, chest-drain omission, and urinary-catheter avoidance

These definitions are provided to clarify the distinct interventions that are often collectively referred to as “Tubeless” in the literature. The fully Tubeless pathway represents the combination of non-intubated anesthesia, chest-drain omission, and urinary-catheter avoidance, which is the primary focus of this review unless otherwise specified.

## 2 The physiological basis and rationale of tubeless technology

**Figure 1:**
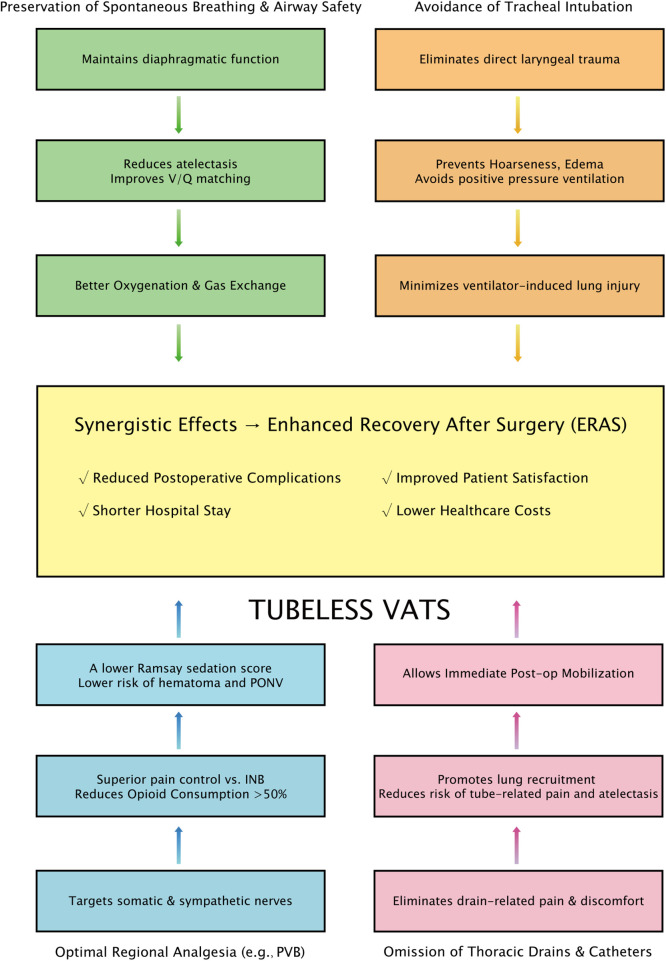
**Physiological mechanisms of Tubeless VATS promoting enhanced recovery after surgery.** The schematic illustrates the four core mechanistic pathways through which the Tubeless technique (characterized by spontaneous ventilation and the avoidance of endotracheal tubes and thoracic drains) synergistically attenuates surgical stress and accelerates postoperative recovery. 1) Preservation of Spontaneous Breathing & Airway Safety: Maintains diaphragmatic function and negative intrathoracic pressure, reducing atelectasis and improving ventilation-perfusion matching. 2) Avoidance of Tracheal Intubation: Eliminates direct airway trauma and prevents ventilator-induced lung injury. 3) Optimal Regional Analgesia: Paravertebral block (PVB) provides superior pain control, reducing opioid consumption and its associated side effects. 4) Omission of Thoracic Drains & Catheters: Eliminates drain-related pain and discomfort, facilitating immediate postoperative mobilization. The convergence of these pathways leads to improved oxygenation, reduced complications, shorter hospital stay, and enhanced patient satisfaction, fully aligning with the principles of Enhanced Recovery After Surgery (ERAS).

Tubeless technology, as discussed in this review, encompasses a range of strategies aimed at eliminating traditional invasive lines, including non-intubated anesthesia (using laryngeal masks or preserving spontaneous breathing), omission of chest drainage tubes, and avoidance of urinary catheters. The successful implementation of Tubeless technology stems from its profound optimization of perioperative respiratory and pain-related physiological processes: the reduction of respiratory interference and the precise regulation of anaesthetic analgesia strategies collectively form the core mechanism that promotes rapid recovery in patients with pulmonary nodules (Figure 1). A deep understanding of its physiological principles is key to grasping the essence and clinical application of Tubeless technology.

### 2.1 Mechanisms for reducing iatrogenic injury

#### 2.1.1 Airway protection: comparative injury profiles of laryngeal mask airway vs. endotracheal tube

While traditional endotracheal intubation (ETT) enables ventilation control, it is inherently an invasive and high-risk procedure. The act of intubation directly triggers sympathetic nervous system activation, causing sudden blood pressure spikes and accelerated heart rate, posing significant threats to patients with concomitant cardiovascular conditions (8). There is also a risk of esophageal misplacement going unrecognized or causing secondary injury in patients with concomitant cervical spine injuries. Tracheal intubation carries risks including aspiration, hemodynamic fluctuations, esophageal misplacement, and exacerbation of cervical spine injury (9).

Furthermore, the use of neuromuscular blockers and sedative-analgesic agents may adversely affect the circulatory system (10). In contrast, Tubeless technology abandons the traditional tracheal intubation approach in favor of laryngeal mask ventilation or maintaining spontaneous breathing throughout the procedure, thereby achieving dual optimization at both structural and functional levels. Structurally, it directly avoids the physical damage to the airway caused by tracheal tubes.

#### 2.1.2 Preservation of spontaneous breathing: impact on early postoperative pulmonary function recovery (SpO_2_, EtCO_2_)

At the functional level, preserving spontaneous breathing maintains active diaphragmatic contraction, which helps sustain negative intrathoracic pressure. This not only aligns more closely with physiological conditions and promotes alveolar re-expansion to reduce atelectasis, but also facilitates optimization of the ventilation/perfusion ratio, thereby reducing the risk of pulmonary function impairment. Research by Zhengcheng Liu et al. provides direct evidence for this: during thoracoscopic wedge resection, patients in the Tubeless group showed comparable blood oxygen saturation (SpO₂: 96.1 ± 2.8% vs. 96.0 ± 2.6%) and end-tidal carbon dioxide pressure (EtCO₂: 44.9 ± 4.8 mmHg vs. 45.1 ± 5.3 mmHg) to the conventional intubation group (11). Although these differences reached statistical significance, the absolute differences were small and their clinical relevance is limited. Other studies have corroborated this finding and observed a more pronounced advantage in EtCO₂ control for the Tubeless group: The mean end-tidal carbon dioxide partial pressure in the Tubeless group was 10 mmHg lower than in the conventional intubation group (SpO_2_: 96.8 ± 3.2% vs. 96.2 ± 3.9%; EtCO_2_: 38.6 ± 10.8 mmHg vs. 43.7 ± 8.1 mmHg) (12). These favorable EtCO₂ results, however, should be interpreted with caution. They were obtained under optimized conditions in highly selected patients (ASA I–II, normal baseline pulmonary function, peripheral small nodules) with relatively low artificial pneumothorax pressures. Therefore, these findings do not imply that Tubeless techniques universally improve CO_2_ elimination; rather, they reflect the best-case scenario achievable with strict patient selection and meticulous intraoperative management. These data collectively demonstrate that Tubeless technology minimizes pulmonary function impairment by simultaneously preventing airway trauma at its source and preserving spontaneous breathing physiology, thereby ensuring ventilation safety. This dual mechanism provides a robust physiological foundation for accelerating patient recovery postoperatively. For a detailed discussion of hypercapnia as a potential intraoperative risk, see Section 4.4.2.

### 2.2 Paravertebral block for attenuating the pain reflex

The successful implementation of Tubeless technology relies on safe and effective regional anesthesia techniques. Paravertebral block (PVB) is widely used in Tubeless thoracic surgery, particularly pulmonary nodule resection, due to its excellent analgesic efficacy (13, 14). By precisely injecting local anesthetics into the paravertebral space, it blocks the dorsal and ventral roots of the corresponding spinal nerves (including sympathetic nerve fibers), thereby creating an extensive analgesic zone that aligns with the surgical incision.

#### 2.2.1 Pain relief effectiveness and safety benefits

**Figure 2:**
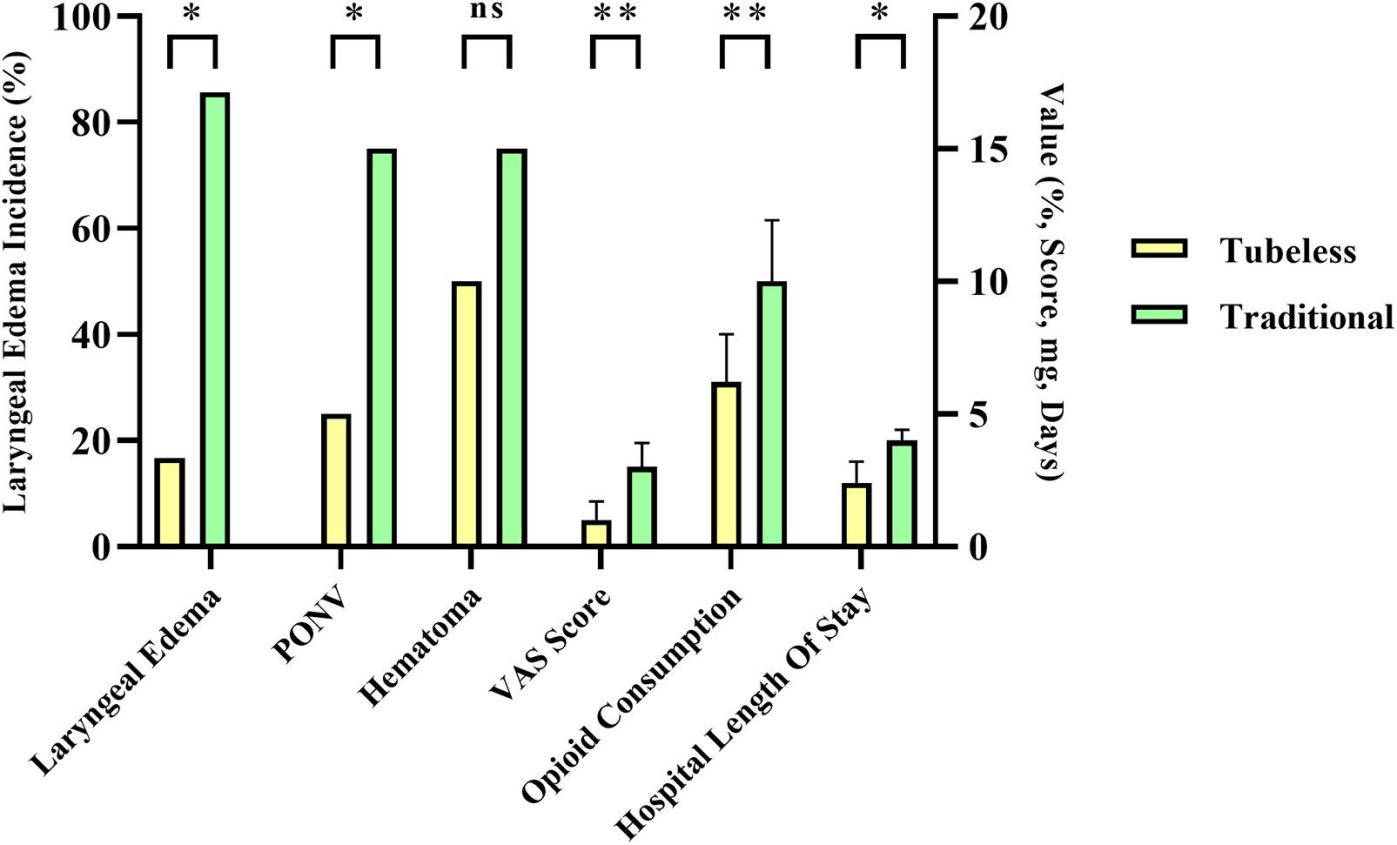
**Comparative outcomes of Tubeless versus traditional intubated VATS for pulmonary nodules.** Data are presented as the incidence rate (percentage) for complication outcomes (Laryngeal Edema, PONV, Hematoma) or as mean ± standard deviation for continuous variables (VAS Score, Opioid Consumption, Hospital Length of Stay). Error bars represent standard deviation. *P* values (as reported in the original studies) are indicated above the brackets: *p < 0.05, **p < 0.01. ns denotes not significant (*p* ≥ 0.05). Source, sample size, and study design for each outcome: Laryngeal edema: from Tanaka et al. (**35**), prospective cohort subset analysis (Tubeless: *n*=6; Traditional: *n*=7); PONV, Hematoma, Opioid use, Hospital stay: from Xia et al. (**15**), randomized controlled trial (*n*=20 per group); VAS score: from Liu et al. (**11**), prospective cohort study (*n*=55 per group). No formal meta-analysis was performed. *PONV*, postoperative nausea and vomiting; *VAS,* visual analog scale.

Compared to intercostal nerve block (INB), PVB demonstrates significant advantages in analgesic efficacy and safety. Firstly, it provides superior analgesia. A study of patients undergoing thoracoscopic isolated pulmonary nodule resection showed that the PVB group exhibited better sedation control at 4 and 8 h postoperatively compared to the INB group, with reduced Ramsay sedation scores at both time points(lower Ramsay score indicates lighter sedation): The PVB group decreased scores from 3.1 to 2.9 at 4 h post-op and from 3.0 to 2.5 at 8 h post-op, with an average difference of 0.35 points between groups. Additionally, the first report of postoperative pain occurred significantly later in the PVB group compared to the INB group (15). This indicates that PVB effectively blocks surgical trauma-induced stimuli at the source and controls acute postoperative pain, laying the foundation for early postoperative mobility in patients (16). It is worth noting that these analgesic advantages of PVB are independent of chest-drain omission. Even when a chest tube is placed, PVB offers superior pain control compared to other techniques, such as INB. Owing to its superior analgesic efficacy, patients in the PVB group experienced significantly accelerated recovery, with their average hospital stay reduced by approximately 1.6 days compared with the INB group (15). Secondly, PVB is associated with fewer adverse effects. The incidence of postoperative nausea and vomiting (PONV) was significantly lower in the PVB group compared to the INB group (5% vs. 15%), as was the incidence of local hematoma (10% vs. 15%) (Figure 2) (15), directly enhancing patient safety. Recent evidence from the OPtriAL Study further corroborates these findings, demonstrating that PVB provides non-inferior analgesia compared to thoracic epidural analgesia with fewer side effects (17).

Other regional analgesic techniques, including serratus anterior plane block (SAPB), erector spinae plane block (ESPB), and thoracic epidural analgesia (TEA), have been described in thoracic surgery. However, comparative studies specifically within Tubeless VATS are scarce, and PVB remains the most extensively investigated technique in this context (14, 17).

#### 2.2.2 Reducing of opioid dependence

Last but most crucially, paravertebral block may reduce opioid dependency: the PVB group consumed 50% fewer opioids within 24 h postoperatively compared to the INB group (6.2 ± 1.8 mg vs. 10.0 ± 2.3 mg) (Figure 2) (15). PVB reduces the need for opioid dependence, thereby effectively avoiding drug-related systemic side effects and the risk of complications, providing patients with stable conditions for recovery (16). Therefore, PVB plays an important role in facilitating the effective implementation of Tubeless technology and supporting enhanced recovery. Current evidence, primarily from randomized trials, suggests favorable analgesic efficacy and safety profiles compared with intercostal nerve blocks (15); however, larger studies are warranted to confirm its superiority. Its precision, efficiency, and safety make it a valuable component of Tubeless treatment for pulmonary nodules and ERAS protocols.

Potential risks associated with Tubeless techniques must also be acknowledged. Hypercapnia is a common physiological consequence, particularly when spontaneous ventilation is maintained under artificial pneumothorax; reported incidence varies but can reach 100% in some cohorts. Hypoventilation may also occur, requiring careful monitoring of respiratory rate and tidal volume. Furthermore, intraoperative conversion to intubation is required in approximately 4%–10% of cases, most often due to refractory hypoxemia, severe hypercapnia, or intraoperative bleeding. These risks underscore the importance of meticulous patient selection, continuous monitoring, and a well-established emergency protocol.

In summary, the physiological superiority of Tubeless technology does not stem from a single technical breakthrough, but rather from the synergistic integration of two pillars: structure-function dual-optimized respiratory management and precise-efficient regional analgesia. From preserving spontaneous breathing by avoiding airway injury to utilizing PVB to block traumatic stress and reduce opioid dependency, this technology forms a comprehensive physiological system. It minimizes disruption to pulmonary function and surgical stress, thereby establishing a robust theoretical foundation for patients' rapid postoperative recovery.

## 3 Clinical practice of tubeless technique

### 3.1 Evolution and current clinical applications of tubeless technology

Since its inception, Tubeless technology has been increasingly adopted in specialized thoracic surgery centers worldwide, accumulating substantial evidence and representing a notable advancement in the minimally invasive treatment of lung nodules. Originating from preliminary explorations in non-intubated thoracoscopic surgery, the technique was systematically proposed and its clinical pathway established by Professor He Jianxing's team. Its core objective is to minimize perioperative iatrogenic injury and physiological stress responses (12). In 2017, Yang and Li et al. reported 30 and 34 successful cases of pulmonary nodule resection using Tubeless technology, respectively, further validating its feasibility and safety while providing early clinical evidence for its broader adoption (12, 18). The 2021 international consensus further clarified patient selection criteria (Table 2): recommending Tubeless application for patients with an ASA score ≤III, BMI < 30 kg/m^2^, good cardiopulmonary function (FEV1% > 50%), and anticipated surgery duration <2 h; while severe pleural adhesions, coagulation disorders, difficult airways, and hypercapnia (PaCO_2_ > 50 mmHg) are listed as contraindications (6). However, a 2022 study specifically examining non-intubated VATS lung biopsy in obese patients (mean BMI: 34.1 kg/m^2^) found no significant differences in complications or 30-day mortality compared to non-obese controls (58), suggesting that for minor procedures such as wedge biopsy, BMI alone may not be an absolute barrier. This consensus marks Tubeless technology's entry into a new phase of standardized yet individualized application.

**Table 2:** Patient selection criteria and institutional requirements for Tubeless VATS.

Category	Criteria	Comments
Ideal Indications	Nodule characteristics: Peripheral location, size ≤ 3 cm, GGO-predominant	Facilitates wedge resection
		Reduces technical difficulty
	Pulmonary function: FEV₁ ≥ 50% predicted, DLCO ≥ 60%	Ensures adequate respiratory reserve during spontaneous ventilation
	General status: ASA class I-II, BMI < 30 kg/m²	Minimizes risks associated with sedation and potential conversion
Relative Contraindications	BMI: 30–35 kg/m²	May be feasible with experienced team
		Case-by-case decision
	**COPD:** Mild-to-moderate (GOLD 1–2)	May tolerate mild hypercapnia
		Closer monitoring required
	**Previous surgery:** Prior ipsilateral thoracic surgery without confirmed dense adhesions	Can be considered in the absence of dense pleural adhesions
Absolute Contraindications	**Nodule characteristics:** Central location requiring lobectomy or systematic lymph node dissection	Tubeless not recommended
		Traditional intubated VATS preferred
	**Coagulopathy:** INR > 1.5, platelet count < 80,000/μL	Increases risk of bleeding
		Requires careful risk-benefit assessment
	**Comorbidities:** Severe COPD (GOLD stage 3–4), severe pulmonary hypertension	May not tolerate hypercapnia or transient hypoxemia
	**Airway:** Known difficult airway	Emergency conversion may be challenging or delayed
Anesthetic requirements	**Team expertise:** Experienced anesthesiology team	Critical for patient safety
	**Equipment:** Capability for lateral-position intubation; DLT or bronchial blocker available	Enables safe emergency conversion
	**Monitoring:** Real-time EtCO₂ monitoring	Allows early detection of hypercapnia
Surgical requirements	**Surgeon expertise:** Experience in thoracoscopic surgery	Reduces operative time and complications
	**Intraoperative capability:** Ability to manage bleeding without tube	Essential for Tubeless success
	**Pathology support:** Intraoperative frozen section available	Helps avoid unnecessary lobectomy
		Preserving Tubeless candidacy
Institutional prerequisites	**Protocol:** Emergency conversion protocol in place	Ensures team preparedness
	**Communication:** Clear multidisciplinary communication	Surgeon-anesthesiologist coordination
	**Equipment:** Emergency intubation equipment (DLT, bronchial blocker, video laryngoscope)	Readiness for unexpected conversion

Data are synthesized from expert consensus and clinical studies (6, 12, 48). Ideal indications represent optimal candidates; relative contraindications require case-by-case assessment; absolute contraindications preclude Tubeless use. Anesthetic, surgical, and institutional prerequisites are essential for safe implementation. ASA, American society of anesthesiologists; BMI, body mass index; COPD, chronic obstructive pulmonary disease; DLT, double-lumen tube; FEV₁, forced expiratory volume in 1 second; GGO, ground-glass opacity; INR, international normalized ratio.

As the technical concept continues to mature, the application of Tubeless technology has gradually expanded. Multiple studies report that the cumulative number of Tubeless surgeries worldwide has been steadily increasing, with thousands of cases reported from China, Italy, the United States, Japan, and several European countries. This suggests that the technique is increasingly recognized as a feasible minimally invasive approach in selected high-volume centers. Mainland China has contributed the majority of reported cases, reflecting the region's active role in the early adoption and refinement of Tubeless techniques (19).

Despite these promising results, the widespread application of Tubeless techniques faces several challenges. Potential intraoperative risks include hypercapnia, hypoxemia, and the need for emergency conversion to intubation, with reported conversion rates of approximately 4%–10%. Moreover, a substantial learning curve is required for both surgeons and anesthesiologists to safely perform Tubeless procedures, and current evidence largely originates from experienced centers. Therefore, rigorous patient selection, structured training, and established emergency protocols are essential before broader implementation.

Furthermore, the applicability of this technology has expanded from early-stage wedge resection to include anatomic segmentectomy, pleural biopsy, bullectomy, and sympathectomy, reflecting its strong adaptability and clinical value (6). For instance, one study reported that among 91 patients undergoing Tubeless procedures (including sympathectomy, bullectomy, and mediastinal tumor resection), only 2 experienced postoperative complications, suggesting a favorable safety profile for treating other conditions (7).

### 3.2 Personalized selection of surgical techniques

The successful implementation of the Tubeless technique is also closely tied to the specific surgical approach selected. The complexity of the procedure, its anticipated duration, and the anatomical scope directly influence the difficulty of managing physiological compensation and risk control in the Tubeless setting. Therefore, establishing surgical selection principles aligned with the Tubeless philosophy is crucial to ensuring its safety and enabling its advantages to be realized.

For peripheral pulmonary nodules (especially ground-glass nodules ≤ 2 cm), thoracoscopic wedge resection is preferred due to its simplicity and favourable preservation of pulmonary function (20). This procedure maximizes lung parenchyma preservation by precisely localizing the lesion while ensuring adequate margins. Its core advantages include short operative time and low complication risk (21). However, selection must follow individualized principles: Anatomical segmentectomy has unique value when the nodule is centrally located within a segment or when intraoperative frozen-section analysis indicates minimally invasive adenocarcinoma. Brunelli et al.'s study, based on retrospective data from a large registry, suggested that segmentectomy may reduce the incidence of cardiopulmonary complications and 30-day mortality compared to lobectomy in elderly or comorbid patients (relative risk ratios of 0.71 and 0.65, respectively) (22). However, these findings should be interpreted with caution due to potential selection bias and residual confounding inherent in retrospective analyses. Prospective randomized trials are needed to confirm the comparative effectiveness of segmentectomy vs. lobectomy in the context of Tubeless surgery. When wedge resection or segmentectomy is performed for suspected lung cancer, several principles apply. Surgical margins should be negative and preferably ≥2 cm or equal to the nodule diameter. Intraoperative frozen section is recommended to confirm margins and rule out upstage invasive disease. The consolidation-to-tumor ratio (C/T ratio) helps guide resection extent: C/T ≤ 0.25 favors wedge resection, whereas C/T > 0.5 suggests invasive adenocarcinoma and may warrant segmentectomy or lobectomy. Solid component size (rather than total nodule size) predicts occult lymph node metastasis; lesions with a solid component >2 cm may require systematic nodal evaluation. For lymph node management, wedge resection does not require dissection; for segmentectomy, hilar and mediastinal sampling is advised when the solid component exceeds 1 cm. Finally, long-term oncological safety of Tubeless sublobar resection remains insufficiently established. Additionally, potential limitations of segmentectomy must be acknowledged: inadequate lymph node dissection may increase the risk of local recurrence. Therefore, strict adherence to indications is essential, and intraoperative lymph node sampling or dissection should be integrated to balance prognosis and trauma (22).

In summary, Tubeless technology is not merely a tube removal technique but a deep integration of evidence-based medicine and perioperative management. By establishing individualized surgical plans, it demonstrates significant value in enhancing patient comfort, accelerating recovery, constituting a notable advancement in thoracic perioperative care.

## 4 Advantages of tubeless technology: comparison with traditional thoracic surgical access

### 4.1 Precision and personalisation in drug combination therapy

#### 4.1.1 Limitations of traditional approaches: reliance on high-dose opioid medications increases the risk of postoperative nausea and vomiting

Traditional thoracic surgery anesthesia regimens heavily rely on high doses of long-acting opioids such as fentanyl (half-life 2–4 h) and the neuromuscular blocker rocuronium bromide. While this approach provides stable anaesthetic depth during surgery, the associated risk of postoperative complications cannot be overlooked. The extensive use of opioids leads to postoperative nausea and vomiting rates as high as 40% (23). It also suppresses respiratory centre function, leading to reduced tidal volume and respiratory rate, thereby increasing the risk of postoperative hypoxemia. This poses particular danger, particularly in patients with preexisting pulmonary impairment (24). Furthermore, rocuronium bromide—a neuromuscular blocker routinely used to facilitate endotracheal intubation—carries the risk of inducing postoperative residual muscle weakness, including respiratory distress and the need for reintubation after extubation. Its induced loss of systemic muscle tone directly decreases functional residual capacity (FRC) and causes diaphragmatic displacement. Consequently, atelectasis occurs in nearly 90% of patients, severely impairing gas exchange and increasing the risk of pulmonary infection (23).

#### 4.1.2 Novel drug combination strategy: dexmedetomidine, sufentanil, and TCI propofol

To overcome the limitations of traditional anesthesia, Tubeless technology employs an opioid-free anesthesia (OFA) strategy (25). Its core protocol uses a loading dose of dexmedetomidine (1 μg/kg loading dose) to selectively inhibit α2 receptors, thereby blocking adrenergic responses and stabilizing circulation. Combined with sufentanil (0.1–0.2 µg/kg), which has a short context-sensitive half-time of approximately 30 min after brief infusion, to deliver potent analgesia and significantly shorten recovery time. This is further supported by target-controlled infusion (TCI) of propofol (2–3 µg/mL) for precise sedation control (18), enabling individualized and precision-tailored anesthesia protocols for patients (Table 3). This approach also incorporates a pre-lubricated laryngeal mask airway (LMA) with spontaneous breathing retention strategy (FiO_2_ 100%, flow rate 4–5 L/min) to maintain oxygen saturation above 95% (11). Compared to conventional anesthesia, this drug combination offers key advantages: reduced risk of postoperative complications, avoidance of neuromuscular blocker-related atelectasis, accelerated recovery with shorter hospital stays, and stable maintenance of oxygen saturation. It is particularly well-suited for short-duration, minimally invasive procedures such as thoracoscopic lung nodule resection, while also serving as a core practice in the concept of enhanced recovery after surgery (ERAS).

**Table 3:** Comparison of traditional intubated anesthesia vs. Tubeless anesthetic protocols.

Component	Traditional intubated anesthesia	Tubeless anesthesia protocol	Key advantages of tubeless approach
Airway Device	Double-lumen endotracheal tube (DLT)	Laryngeal mask airway (LMA)	Reduces airway trauma
Ventilation Mode	Positive-pressure, one-lung ventilation (OLV)	Spontaneous ventilation (SV)	Preserves diaphragmatic function
Key Agents	Induction: High-dose opioids	Induction/Maintenance: Dexmedetomidine + Sufentanil + TCI Propofol	Reduces PONV and opioid use
	Maintenance: neuromuscular blockers		Reduces VILI
Analgesia	Systemic opioids (postoperative)	Regional block (e.g., PVB)	Superior pain control (VAS score ↓)
			Facilitates early mobilization
Monitoring Focus	Airway pressure, tidal volume, PEEP	EtCO₂, respiratory rate, SpO₂	Non-invasive
			Allows early detection of hypercapnia

Protocols are derived from comparative clinical studies.

PONV, postoperative nausea and vomiting; VAS, visual analog scale; VILI, ventilator-induced lung injury; TCI, target-controlled infusion; PVB, paravertebral block.

### 4.2 Innovation and selection of ventilation strategies

#### 4.2.1 Traditional single-lung ventilation issues: high airway pressure causing lung injury and risk of CO_2_ retention

Additionally, Tubeless technology has made new advances in ventilation strategies. While the one-lung ventilation (OLV) technique used in traditional thoracic surgery provides excellent surgical exposure, its inherent physiological interference and risk of complications cannot be overlooked (26, 27): First, OLV requires higher pressures to maintain, which can easily cause mechanical lung injury. A retrospective analysis of 146 total lung resection cases revealed that postoperative pulmonary edema correlates with elevated airway peak pressure. For every 1 cm H₂O increase in airway peak pressure, the odds ratio for acute lung injury increases by 2.32 (28). Another study confirmed that acute respiratory distress syndrome (ARDS) may occur in 2.9% of patients after lobectomy and 7.9% after total lung resection under single-lung ventilation, with mortality rates as high as 42% and 50%, respectively (29). Second, the increased dead-space volume from single-lung ventilation leads to CO₂ retention, inducing hypercapnia (PaCO₂ > 50 mmHg) and pulmonary hypertension. Consequently, patient selection is restricted: strategies involving single-lung ventilation should be avoided in patients with pulmonary hypertension, right heart failure, or intracranial hypertension (29). Research demonstrates that protective ventilation with low tidal volumes during single-lung ventilation may increase dead space and cause CO₂ retention (hypercapnia). Adjusting the respiratory rate may trigger auto-PEEP and barotrauma, leading to lung injury. This suggests that single-lung ventilation may be an imperfect solution (29).

#### 4.2.2 Tubeless alternatives and their advantages: high-frequency ventilation (HFJV), laryngeal mask airway (LMA)

In response, Tubeless technology has optimized two distinct strategies: high-frequency jet ventilation (HFJV) and laryngeal mask airway (LMA) ventilation. Gomez-Ríos et al. explicitly identified HFJV as a valuable oxygenation strategy. Its principle is to deliver small tidal volumes at high frequency (100–1500 breaths per minute) through a thin catheter, thereby maintaining minimal alveolar pressure while preserving spontaneous breathing (30). Data indicate that transcutaneous high-frequency jet ventilation (TCHFJV) achieves a 90% surgical success rate with low complication rates. It provides adequate surgical visualization and ventilation without requiring endotracheal intubation during surgery, with postoperative subcutaneous emphysema occurring in only 8.4% of cases and pneumothorax in just 1% (30).

As the core component of Tubeless technology, the laryngeal mask airway (LMA) can be used with HFJV devices to avoid tracheal intubation, preserving a larger airway diameter for stable ventilation (30). Research by Yuying Liu et al. demonstrated that the LMA offers unique advantages in cases where tumors are close to the glottis or where post-intubation stenosis occurs, as the supraglottic region remains unaffected by narrowed airways (31). Compared with traditional single-lung ventilation, which may cause mechanical lung injury and abnormal oxygenation parameters, the optimized dual-ventilation strategies enabled by Tubeless technology have successfully liberated the surgical field without sacrificing lung protection.

Tubeless technology addresses limitations of conventional thoracic anesthesia through dual innovations: an innovative drug combination (OFA strategy) and more physiologically aligned ventilation modes (HFJV/LMA). Its core advantages lie in achieving personalised, precise anesthesia through receptor modulation and the metabolism of ultra-short-acting agents, while preserving spontaneous breathing to maintain intact pulmonary physiology and normal oxygenation parameters. This enables minimally invasive surgery to truly enter the Tubeless era. This approach not only aligns with the principles of enhanced recovery but also establishes a promising approach for lung-protective anesthesia in clinical practice.

### 4.3 Evidence supporting reduced complications

#### 4.3.1 Laryngeal edema incidence: laryngeal mask vs. endotracheal intubation

The clinical advantages of Tubeless technology extend beyond procedural convenience to include significant improvements in patient outcomes and reduced complication risks (32, 33). Regarding airway safety, multiple studies confirm its clear superiority over traditional endotracheal intubation. Gonzalez-Rivas et al. reported that in a non-intubated VATS cohort, none of the 3 patients experienced postoperative hoarseness or dysphagia, supporting the notion that avoiding invasive airway devices mitigates the risk of postoperative laryngeal edema (34). Atsuko Tanaka et al.'s prospective study provided further compelling evidence: the incidence of laryngeal edema in the tracheal intubation group was 85.7% (6/7), whereas it was only 16.7% (1/6) in the laryngeal mask airway (LMA) group, with a statistically significant difference between groups (*P* = 0.029) (Figure 2). The mechanism is that tracheal intubation primarily induces laryngeal oedema through mechanical injury, thereby increasing postoperative laryngeal resistance. In contrast, the laryngeal mask affects vocal cord muscle balance through neuromodulation, reducing damage to airway structures (35). This finding, although derived from a small study (Tanaka et al., *n* = 7 per group), suggests that LMA may reduce airway trauma. Larger studies are needed to confirm this observation.

#### 4.3.2 Drainage tube-related infection rate: drainage-free vs. traditional groups

In infection control, the no-tube strategy has demonstrated positive outcomes, with the underlying mechanism of this advantage closely linked to the pathophysiological burden associated with traditional chest tube drainage. As an invasive foreign body, the chest tube itself serves as a major activator of inflammatory responses (36). Surgical trauma and mechanical irritation trigger the release of pro-inflammatory cytokines, neutrophil aggregation, and activation. The prolonged presence of the chest tube undoubtedly perpetuates and potentially exacerbates this process. Persistent inflammation not only compromises local tissue defense and repair but also creates a favorable environment for bacterial colonization and infection (37).

Data from Shun-Mao et al. provide strong support for the advantages of the no-tube strategy: patient satisfaction with surgical incisions reached 90% (27/30) in the no-tube group, significantly higher than the 73.3% (22/30) in the traditional tube group (12). This improvement stems not only from the marked reduction in pain and discomfort caused by the drainage tube but also directly correlates with the diminished local inflammatory response. Another study reported 1 case (2.3%) of pulmonary infection in the traditional chest tube drainage group, with none in the no-drainage group. Although the difference between groups did not reach statistical significance due to sample size limitations (*P* = 1.000), the observed trend toward zero infections holds potential clinical implications. This also suggests that larger sample sizes are needed to validate its anti-infective benefits (38).

Tubeless technology optimizes perioperative management in thoracic surgery by eliminating multiple invasive accesses, including endotracheal intubation, postoperative chest drainage tubes, and urinary catheters (39). The strategy of removing postoperative chest tubes not only avoids tube-related pain—manifested as an average reduction of 0–2 points in VAS scores (Table 4) (11), but also promotes lung re-expansion by maintaining negative pleural pressure, reducing atelectasis risk. Additionally, improved pain control (e.g., a 50% reduction in opioid use) combined with earlier enteral nutrition initiation contributes to shorter hospital stays (40). Finally, from a medical economics perspective, Tubeless technology demonstrates apparent cost-effectiveness. Eliminating tube placement shortens the surgical turnaround time by 20%, reducing the overall procedure duration to 6–40 min, with an average of 16.73 ± 10.59 min (39). Simultaneously, the decrease in postoperative complications lowers the reintervention rate. This comprehensive optimization reduces overall healthcare costs and improves the efficiency of medical resource utilization.

**Table 4:** Comparative analysis of perioperative outcomes between Tubeless and traditional intubated VATS.

Outcome measure	Study design	Tubeless group	Traditional group	*P* value	Notes
Laryngeal edema	Prospective cohort (subset analysis)	1/6 (16.7%)	6/7 (85.7%)	0.029	^a^
PONV	RCT	1/20 (5.0%)	3/20 (15.0%)	0.024	
Hematoma	Prospective cohort	2/20 (10.0%)	3/20 (15.0%)	0.382	
VAS Score (24 h)	RCT	1.0 ± 0.7 (*n* = 55)	3.0 ± 0.9 (*n* = 55)	<0.01	^b^
Opioid Use (mg, ME)	RCT	6.2 ± 1.8 (*n* = 20)	10.0 ± 2.3 (*n* = 20)	<0.01	
Hospital Stay (days)	RCT	2.4 ± 0.8 (*n* = 20)	4.0 ± 0.4 (*n* = 20)	0.026	

Data are presented as number of events/number of patients assessed (*n*/*N*, %) for dichotomous outcomes or mean ± standard deviation for continuous variables. *P* values are as reported in the original cited studies. No formal meta-analysis was performed; data are synthesized from individual studies for descriptive comparison. Study designs: Laryngeal edema from a prospective cohort subset analysis (35); PONV, hematoma, opioid use, and hospital stay from a randomized controlled trial (15); VAS score from a prospective cohort study (11).

PONV, postoperative nausea and vomiting; VAS, visual analog scale; ME, morphine equivalents.

a Data on laryngeal edema were derived from a study subset specifically designed for the comparative assessment of laryngeal mask vs. endotracheal intubation.

b Data on VAS scores were derived from a study subset specifically designed for the comparative assessment of the Tubeless group vs. the chest drainage group.

### 4.4 Potential risks and management

#### 4.4.1 Overview of intraoperative risks

Several intraoperative adverse events may occur during Tubeless VATS (Table 5). These include hypercapnia, hypoxemia, coughing (triggered by mediastinal manipulation or bronchial irritation), mediastinal shift (due to open pneumothorax), aspiration (of gastric contents or blood), bleeding (from pulmonary vessels or chest wall), dense pleural adhesions (limiting lung collapse), and inadequate analgesia (leading to patient movement or respiratory distress). Conversion to intubated anesthesia should be viewed as a planned safety measure rather than a failure, and a predefined conversion protocol is essential. The following subsections discuss the most common risks and their management.

**Table 5:** Intraoperative risks and management during Tubeless VATS.

Risk	Prevention	Management
Hypercapnia	Limit pneumothorax pressure (lowest effective level)	Increase respiratory rate
		Manual assist
Hypoxemia	Preoxygenate (FiO₂ 100%)	Increase FiO₂
		Convert if refractory to oxygen therapy
Coughing	Topical airway anesthesia; vagal block	Deepen sedation
		Pause manipulation
Mediastinal shift	Monitor position clinically	Reduce insufflation
	Limit CO₂ flow	Convert if severe
Aspiration	Standard preoperative fasting	Suction
		Convert to intubation
Bleeding	Careful dissection	Compression
	Energy devices	Convert for major bleeding
Dense adhesions	Preoperative imaging assessment	Convert early if lung collapse inadequate
Inadequate analgesia	Effective PVB	Supplement with short-acting opioid
Emergency conversion	Predefined criteria	Regard as necessary intervention for patient safety
	Equipment ready	

Common intraoperative risks and management during Tubeless VATS. Conversion to intubated anesthesia should be viewed as a necessary intervention for patient safety.

PVB, paravertebral block.

#### 4.4.2 Hypercapnia: adjust ventilation parameters (EtCO_2_ monitoring)

The apparent discrepancy between favorable EtCO₂ outcomes in selected studies and the high incidence of hypercapnia in routine practice can be explained by differences in patient selection, anesthetic protocols, and surgical conditions. Studies reporting lower EtCO₂ in Tubeless groups typically enrolled highly selected patients (ASA I–II, normal pulmonary function, peripheral small nodules) with low artificial pneumothorax pressures and short operative times. In contrast, the studies cited below include a broader patient population, often requiring higher pneumothorax pressures for adequate lung collapse or involving longer procedures, both of which increase CO₂ retention. Therefore, the favorable EtCO₂ data should not be generalized to all Tubeless cases; hypercapnia remains a common and expected physiological challenge that requires active monitoring and management.

Hypercapnia represents the most common physiological complication (11): Studies indicate that the incidence of intraoperative hypercapnia (PaCO₂ > 45 mmHg) reached 100% in the spontaneous ventilation (SV) group, significantly higher than the 0% rate in the mechanical ventilation (MV) group (*P* < 0.001). PaCO₂ was significantly higher in the spontaneous ventilation (SV) group than in the mechanical ventilation (MV) group (64.14 ± 9.30 mmHg vs. 39.81 ± 3.34 mmHg), representing an average increase of approximately 25 mmHg. Similarly, EtCO₂ values showed a comparable elevation in the SV group (62.30 ± 5.53 mmHg vs. 36.77 ± 2.94 mmHg), with an average difference of 26 mmHg. These data effectively demonstrate that the spontaneous ventilation group is more prone to hypercapnia (41), a phenomenon attributed to the dual suppression of minute ventilation by artificial pneumothorax and opioid medications (42). Concurrently, PaCO₂ and EtCO₂ values in the spontaneous ventilation group showed a high correlation (64.14 ± 9.30 vs. 62.30 ± 5.53; *R* = 0.96, *P* < 0.001). This indicates that EtCO₂ should be utilized as a non-invasive monitoring parameter to adjust respiratory rate and tidal volume, thereby maintaining PaCO₂ within physiological ranges (42).

#### 4.4.3 Emergency intubation: risk prediction (common in intraoperative bleeding, deteriorating oxygenation)

**Figure 3:**
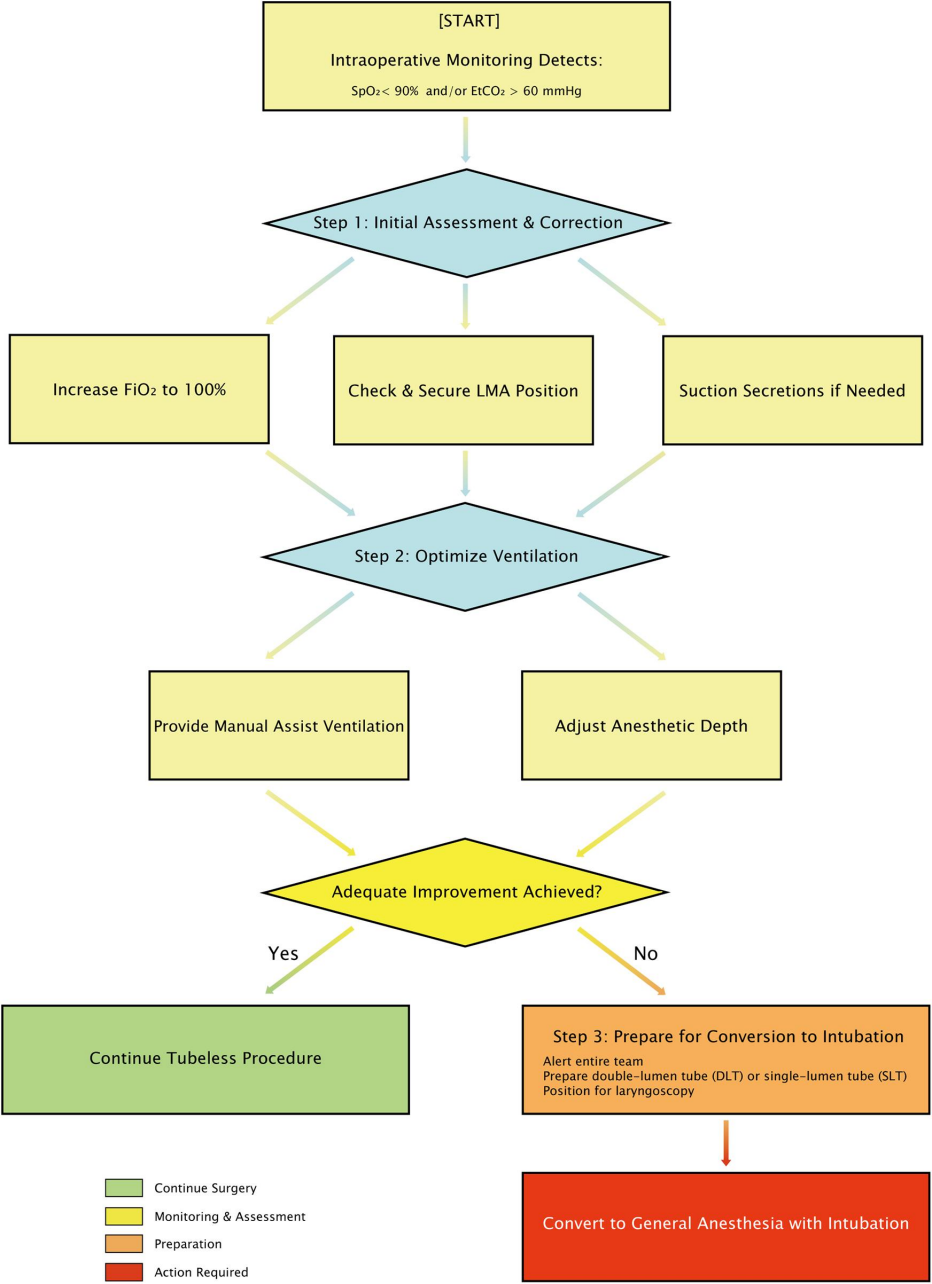
**Intraoperative management algorithm for hypoxemia or hypercapnia during Tubeless VATS**. This clinical decision pathway guides the stepwise response to a decline in oxygen saturation (SpO₂ < 90%) and/or a rise in end-tidal carbon dioxide (EtCO₂ > 60 mmHg). Initial maneuvers focus on correcting reversible causes. “Adequate improvement” was defined as SpO₂ ≥90% and EtCO₂ ≤60 mmHg within 2 minutes of initial corrective measures. If these targets are not met, conversion is recommended. The algorithm mandates preparation for and execution of conversion to conventional intubated general anesthesia to ensure patient safety. The proposed thresholds are derived from clinical experience and institutional protocols; evidence-based validation from prospective trials is currently lacking.

Emergency intubation should be regarded as a safety maneuver rather than a procedural failure. Its overall incidence is approximately 4%–10% (including a rate of about 4.1% in Tubeless surgery for lung cancer) (43). Its risk factors primarily stem from severe intraoperative physiological imbalances, including refractory hypoxemia, severe hypercapnia unresponsive to corrective therapy, and urgent situations such as active bleeding during surgery. Research by Weixue et al. indicates that once unresolved intraoperative deterioration of oxygenation or active bleeding occurs, the anesthesia team should immediately switch to endotracheal intubation for airway control (Figure 3) (44). This is not only to ensure reliable ventilation and oxygenation but also to implement lung-protective strategies such as positive end-expiratory pressure (PEEP) and lung recruitment maneuvers under controlled mechanical ventilation, while providing a clear surgical field to manage bleeding sites. Thus, the successful implementation of Tubeless techniques relies heavily on intraoperative collaboration between the anesthesia and surgical teams, along with a well-established and efficient emergency plan for transitioning to endotracheal intubation Min & Seo (45); He & Zhao (46). This transforms a potential risk into a controllable safety event.

Current evidence indicates that Tubeless technology offers significant advantages in reducing airway injury and promoting patient recovery. However, this technique also poses challenges, including hypercapnia and the risk of reintubation. Its successful implementation relies on meticulous intraoperative management and close multidisciplinary team collaboration. Future efforts should focus on establishing standardized protocols to further advance the regulated application of Tubeless technology.

## 5 Current focus and future directions

### 5.1 Key controversies

#### 5.1.1 Safety of non-intubated anesthesia: emergencies such as mediastinal shift

Despite demonstrating significant perioperative advantages, the clinical application of Tubeless anesthesia remains controversial and challenging. Regarding safety, the complexity of intraoperative management in non-intubated anesthesia is particularly prominent (47). Yunpeng et al.'s study revealed that among 174 patients undergoing completely Tubeless surgery, 8 cases (4.6%) required conversion to intubation. Six of these conversions were due to intraoperative bleeding, while two were attributed to severe mediastinal shift. The authors further proposed a weight threshold of approximately 69 kg for mediastinal shift, suggesting that lighter patients (approximately <69 kg) may have a lower risk of mediastinal shift and could be more suitable for non-intubated anesthesia. However, this finding is derived from a single study and should be considered hypothesis-generating rather than definitive guidance for patient selection (48). Conversely, this implies that non-intubated anesthesia carries higher risks for obese or overweight patients, necessitating greater team expertise and comprehensive contingency plans. This concern is echoed in a 2022 Italian national survey, in which a substantial proportion of anesthesiologists considered obesity a contraindication to non-intubated thoracic surgery, and only 38% of centers performed such techniques for parenchymal lung resections (57). We further contend that mediastinal shift is determined by multiple factors—including patient positioning, artificial pneumothorax pressure, and lung collapse rate—rather than solely by weight. We recommend real-time intraoperative monitoring of mediastinal position and oxygen saturation, combined with preoperative CT assessment of pleural cavity volume, to individually set upper limits for pneumothorax pressure and prevent its occurrence. For high-risk patients (e.g., obese, emphysematous), consider adopting an assisted ventilation mode that preserves shallow, spontaneous breathing rather than complete spontaneous breathing to balance surgical visibility and safety. Therefore, the ability to effectively prevent and manage intraoperative emergencies, such as mediastinal shift, is a key criterion for assessing the safe implementation of Tubeless technology and must be addressed to overcome scepticism about its widespread adoption.

#### 5.1.2 Long-term prognosis: insufficient evidence on Tubeless technology's impact on oncological outcomes

Concurrently, there remains a paucity of evidence concerning long-term oncological outcomes. Tommaso and Yaokai explicitly state that while Tubeless technology offers clear short-term recovery advantages, its effects on long-term patient survival, recurrence, and metastasis remain unclear (43, 49). Although existing sample studies do not suggest increased recurrence risk, any definitive conclusions are premature in the absence of large-scale, long-term follow-up data. Therefore, we recommend designing and implementing more rigorous multicenter, prospective cohort studies or randomized controlled trials before this technology is widely adopted. We also encourage systematic reporting of long-term oncological outcomes to establish an evaluation system centered on long-term oncological survival and recurrence-free rates. This approach may enable a scientific and comprehensive assessment of the true value of Tubeless technology in oncological outcomes.

Despite these challenges, ongoing technological innovations offer promising solutions to address the above-mentioned safety concerns and evidence gaps.

#### 5.1.3 Additional considerations

Several additional aspects warrant consideration. First, non-intubated surgery requires explicit patient consent and cooperation, as intraoperative awareness or discomfort may occur. Second, most cost-effectiveness data originate from high-volume Chinese centers; validation in other healthcare systems is needed. Third, comparison with robotic-assisted thoracic surgery (RATS) remains unexplored, although RATS offers precision but at higher cost. Fourth, evidence for elderly, frail, or pediatric populations is scarce. Finally, from a sustainability perspective, Tubeless techniques reduce disposable tube usage, aligning with environmentally conscious surgical practices.

### 5.2 Future optimization

The following directions represent research priorities and conceptual hypotheses rather than established clinical standards. Preliminary evidence and ongoing investigations suggest potential applications, but clinical implementation requires further validation.

#### 5.2.1 Technology integration: ENB preoperative localization + tubeless resection

Potential future directions for Tubeless technology may include: technology integration, intelligent monitoring, and same-day surgery pathways. First, the combined use of Electromagnetic Navigation Bronchoscopy (ENB) and Tubeless resection could enable integrated, precision localization and minimally invasive treatment of pulmonary nodules. Experiments by Lamprecht et al. demonstrated that electromagnetic navigation bronchoscopy combined with rapid on-site cytological evaluation (ROSE) and PET-CT achieved an overall diagnostic rate of 83.9% for pulmonary nodules, maintaining a 75.6% diagnostic rate for nodules ≤20 mm (50). Integrating this precise localization technology into the Tubeless workflow could enable preoperative nodule marking or placement of positioning hooks via ENB, streamlining the intraoperative process (51). This eliminates the need for cumbersome intraoperative CT and its associated radiation exposure, reduces operative time previously spent on localization challenges, and substantially enhances overall procedural efficiency, thereby facilitating safer and more streamlined Tubeless surgeries.

#### 5.2.2 Precision monitoring: AI-driven real-time prediction of reintubation risk

Secondly, AI-based risk prediction systems hold promise in addressing the lag in intraoperative monitoring—such as interventions typically initiated only after oxygenation deterioration (52). The multifaceted risks leading to reintubation demand our attention, making the rational and efficient prediction of intraoperative risks a critical area for future exploration. Research by Muhammad et al. demonstrates that AI can provide early warnings of adverse events, such as deteriorating oxygenation, bleeding, through real-time analysis of EtCO₂ waveforms and thoracoscopic video data, thereby preventing reintubation (53). Future AI applications may further color-code patient risk levels to support intraoperative decision-making, facilitating a shift from reactive management to proactive intervention. For example, a hypothetical Yellow alert (30%–50% risk probability) might automatically optimizes ventilation parameters; an Orange alert (50%–70%) could alerts the surgeon to prepare resuscitation equipment; a Red alert (>70%) could initiates the emergency intubation protocol, thereby significantly enhancing procedural safety. These thresholds are conceptual and await prospective validation.

#### 5.2.3 Day surgery workflow: cost-effectiveness analysis of reduced hospital stay

Finally, the day-surgery model maximizes the economic benefits of Tubeless technology. Shun-Mao noted that although the Tubeless group had shorter hospitalizations than the conventional group, the median length of stay remained substantial at 3 days. Jiangrong et al.'s study demonstrated that the day-surgery model reduced patient stay from 10 h to 6 h, with a further decrease in median hospitalization duration. Readmission rates dropped from 2% to 0.22%, and costs decreased by 35% (54). The feasibility of integrating Tubeless techniques into same-day pathways is further supported by a 2020 retrospective series of 99 consecutive uniportal awake lung biopsies. In this study, all procedures were completed without conversion to general anesthesia, with a diagnostic yield of 98%, mean hospital stay of 1.3 days, and a readmission rate of only 3% with no 30-day mortality (59). These data demonstrate that integrating Tubeless technology into a highly standardized day-surgery pathway enables efficient utilization of medical resources while ensuring patient safety and quality of care (55). Therefore, future efforts should focus on establishing standardized day-surgery clinical pathways based on Tubeless technology and on promoting the transition of simple lung nodule resection procedures toward same-day admission and discharge. This represents not only technological advancement but also an innovative shift in healthcare service delivery models (56).

**Figure 4:**
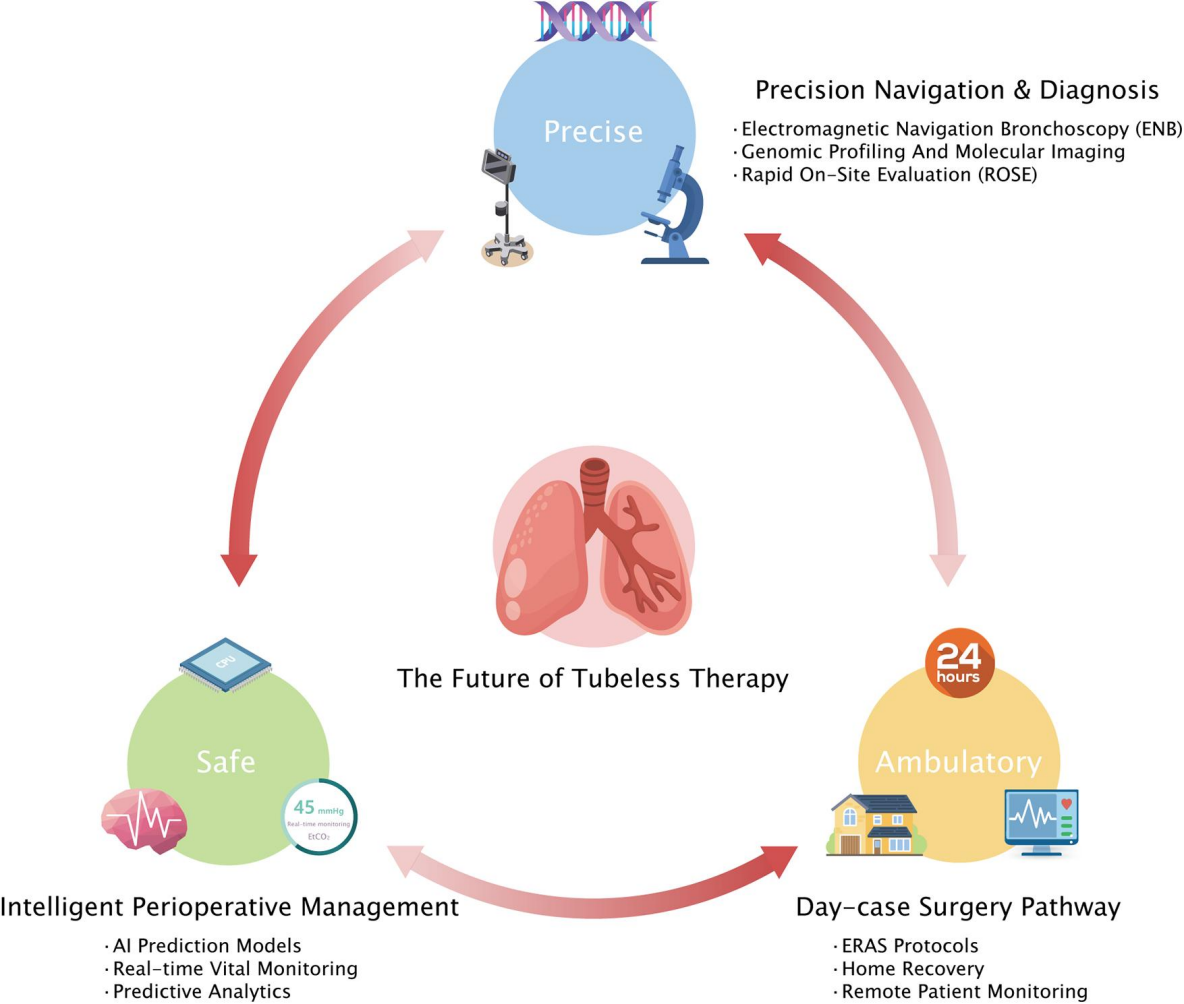
**The integrated innovation framework for the future evolution of Tubeless technology.** This conceptual map illustrates three synergistic innovation drivers poised to advance Tubeless VATS: 1) Precision Navigation & Diagnosis, enhancing preoperative planning and targeting through tools like electromagnetic navigation bronchoscopy (ENB) and molecular imaging; 2) Intelligent Perioperative Management, utilizing artificial intelligence (AI) and real-time vital monitoring for real-time risk prediction; 3) Streamlined Day-Case Surgery Pathways, optimizing patient workflows through enhanced recovery after surgery (ERAS) protocols and remote monitoring. The convergence of these interdisciplinary fields is driving the evolution of Tubeless therapy toward a future paradigm that is more precise, safe, painless, and ultimately suitable for ambulatory settings. The proposed directions are research priorities and conceptual frameworks, not established clinical standards.

Undoubtedly, Tubeless technology is not only present today but could remain prominent in the future. Currently, we should cautiously address issues such as insufficient evidence regarding its safety across different populations and long-term oncological outcomes. Moving forward, integrating technology, implementing intelligent monitoring, and innovating day-surgery pathways may be essential to establishing safer, more efficient, and cost-effective surgical models (Figure 4).

## 6 Conclusions and outlook

Tubeless technology represents a profound integration and significant advancement in the minimally invasive philosophy of thoracic surgery and the practice of enhanced recovery after surgery (ERAS). This review systematically elucidates the complete logical chain of this technology, from its physiological foundations to clinical applications. Its core advantages stem from profound optimization of perioperative physiological processes. Employing laryngeal masks or strategies that preserve spontaneous breathing, it structurally avoids airway injury from endotracheal intubation while functionally maintaining diaphragmatic activity and negative intrathoracic pressure, thereby minimizing mechanical lung injury. Concurrently, precise analgesia—represented by paravertebral blocks—enables superior postoperative pain control and may reduce opioid dependency. This synergistic dual physiological protection system for respiration and analgesia forms the cornerstone of patients' rapid postoperative recovery.

In clinical practice, Tubeless technology offers clear advantages by eliminating multiple tubes, such as endotracheal tubes and chest drainage tubes, thereby reducing complications like laryngeal edema, alleviating postoperative pain, and shortening hospital stays. However, its widespread adoption faces two core controversies: first, managing emergencies such as mediastinal displacement or major hemorrhage in a non-intubated state relies heavily on team experience and contingency plans. Second, there remains a lack of large-scale, long-term follow-up evidence to clarify its oncological outcomes—a critical gap future research must address. Long-term oncological outcomes of Tubeless surgery remain insufficiently established, and available data are limited to small sample sizes or short follow-up periods. Larger prospective studies are needed to confirm oncological safety.

Looking ahead, Tubeless technology may evolve along three primary trajectories: Technological integration, such as combining with electromagnetic navigation bronchoscopy for precise preoperative localization to streamline procedures; Intelligent management, leveraging artificial intelligence for real-time physiological data analysis to predict risks of reintubation, shifting from reactive to proactive intervention; and pathway optimization, integrating Tubeless technology into day surgery models. For day-surgery pathways, anticipated outcome improvements include a reduction in median hospital stay from approximately 3 days to <24 h, a 30-day readmission rate target of <1%, and overall cost savings of 30%–40% based on preliminary evidence. Standardized clinical pathways could maximize resource efficiency while ensuring safety, advancing simple lung nodule surgeries toward same-day admission and discharge.

In summary, Tubeless technology has established itself as a significant minimally invasive option. The next priority is to conduct prospective studies to strengthen its evidence base for safety and oncological efficacy. Through technological innovation and process standardization, this approach may help propel thoracic surgery toward a new era of enhanced safety, precision, and efficiency.
