# Supplementary material for: Research advances of tubeless thoracic surgery for pulmonary nodules: current status and future challenges
Source: Front Surg. 2026 May 25;13:1834893. doi: 10.3389/fsurg.2026.1834893 (PMC13243431; doi:10.3389/fsurg.2026.1834893)
Supplement: Supplementary file 1 [file Datasheet1.zip › Supplementary material presentation/Supplementary_Material.docx]

**
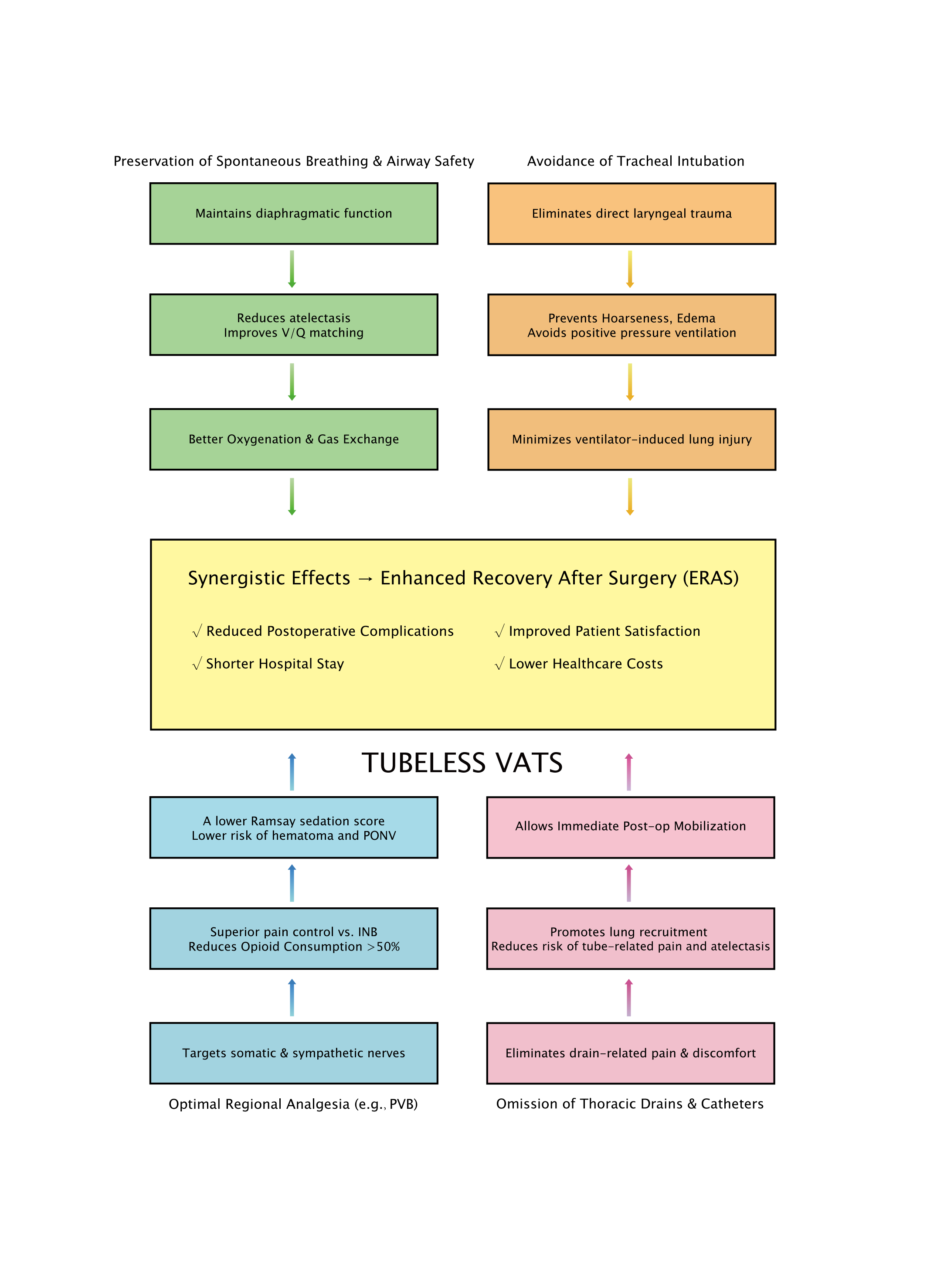
**Supplementary Material

## Supplementary Figure:

**Figure 1. Physiological mechanisms of Tubeless VATS promoting enhanced recovery after surgery.**

The schematic illustrates the four core mechanistic pathways through which the Tubeless technique (characterized by spontaneous ventilation and the avoidance of endotracheal tubes and thoracic drains) synergistically attenuates surgical stress and accelerates postoperative recovery. 1) Preservation of Spontaneous Breathing & Airway Safety: Maintains diaphragmatic function and negative intrathoracic pressure, reducing atelectasis and improving ventilation-perfusion matching. 2) Avoidance of Tracheal Intubation: Eliminates direct airway trauma and prevents ventilator-induced lung injury. 3) Optimal Regional Analgesia: Paravertebral block (PVB) provides superior pain control, reducing opioid consumption and its associated side effects. 4) Omission of Thoracic Drains & Catheters: Eliminates drain-related pain and discomfort, facilitating immediate postoperative mobilization. The convergence of these pathways leads to improved oxygenation, reduced complications, shorter hospital stay, and enhanced patient satisfaction, fully aligning with the principles of Enhanced Recovery After Surgery (ERAS)


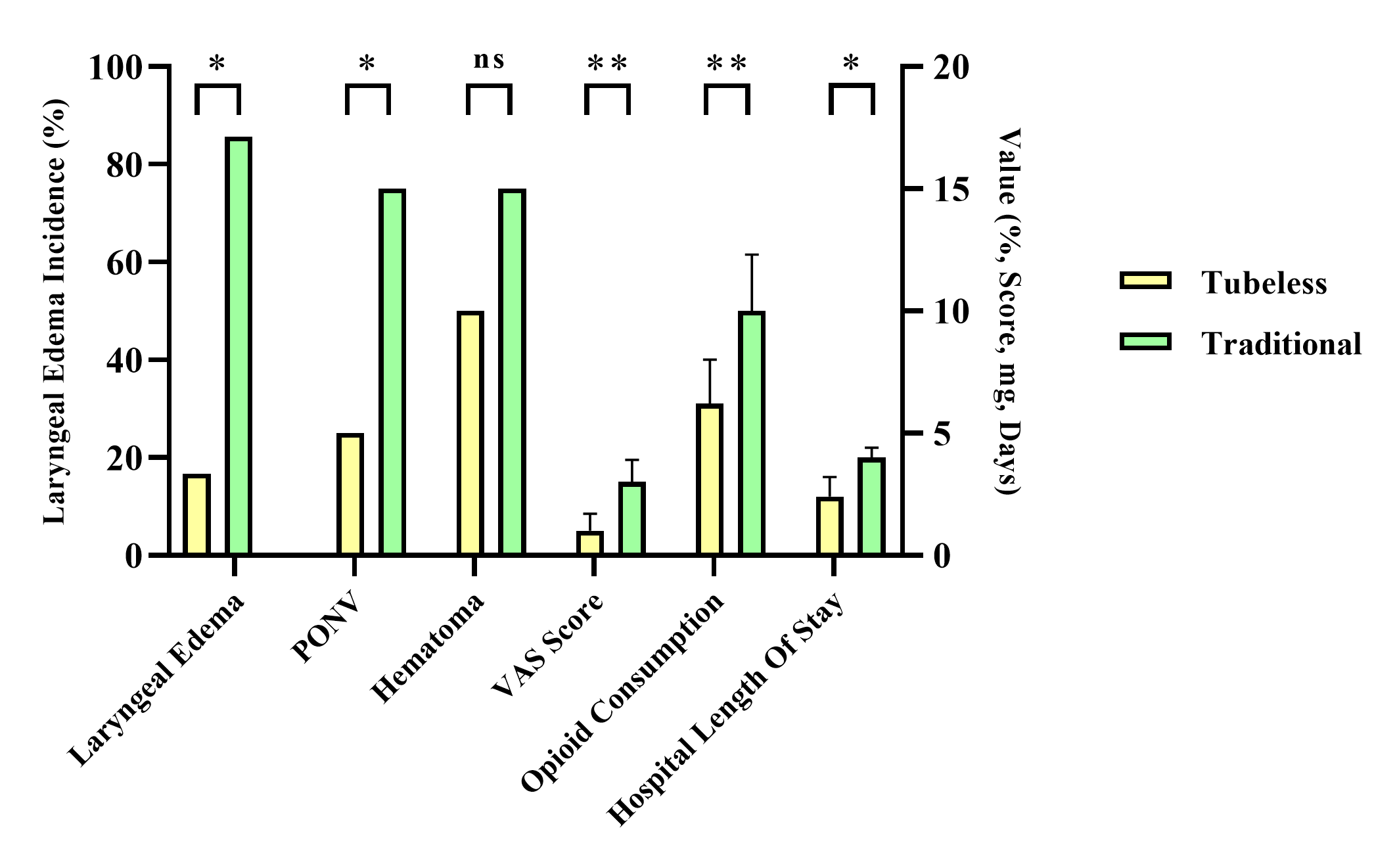


**Figure 2. Comparative outcomes of Tubeless versus traditional intubated VATS for pulmonary nodules.**

Data are presented as the incidence rate (percentage) for complication outcomes (Laryngeal Edema, PONV, Hematoma) or as mean ± standard deviation for continuous variables (VAS Score, Opioid Consumption, Hospital Length of Stay). Error bars represent standard deviation. P values (as reported in the original studies) are indicated above the brackets: *p* < 0.05, **p* < 0.01. ns denotes not significant (p ≥ 0.05).
Source, sample size, and study design for each outcome: Laryngeal edema: from Tanaka et al. [35], prospective cohort subset analysis (Tubeless: n=6; Traditional: n=7); PONV, Hematoma, Opioid use, Hospital stay: from Xia et al. [15], randomized controlled trial (n=20 per group); VAS score: from Liu et al. [11], prospective cohort study (n=55 per group). No formal meta-analysis was performed.
Abbreviations: PONV, postoperative nausea and vomiting; VAS, visual analog scale.


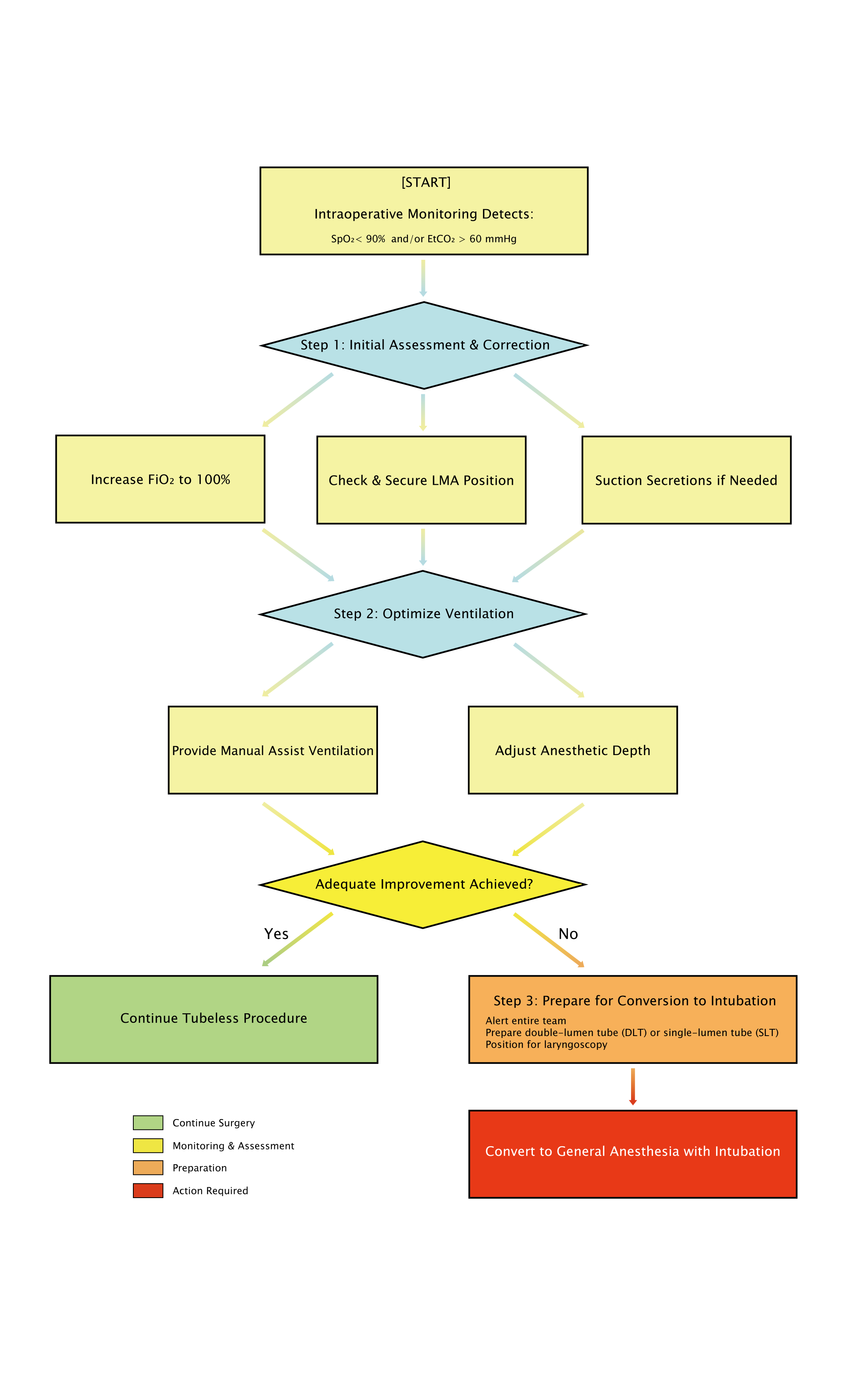


**Figure 3. Intraoperative management algorithm for hypoxemia or hypercapnia during Tubeless VATS.**

This clinical decision pathway guides the stepwise response to a decline in oxygen saturation (SpO₂ < 90%) and/or a rise in end-tidal carbon dioxide (EtCO₂ > 60 mmHg). Initial maneuvers focus on correcting reversible causes. “Adequate improvement” was defined as SpO₂ ≥90% and EtCO₂ ≤60 mmHg within 2 minutes of initial corrective measures. If these targets are not met, conversion is recommended. The algorithm mandates preparation for and execution of conversion to conventional intubated general anesthesia to ensure patient safety.
The proposed thresholds are derived from clinical experience and institutional protocols; evidence-based validation from prospective trials is currently lacking.


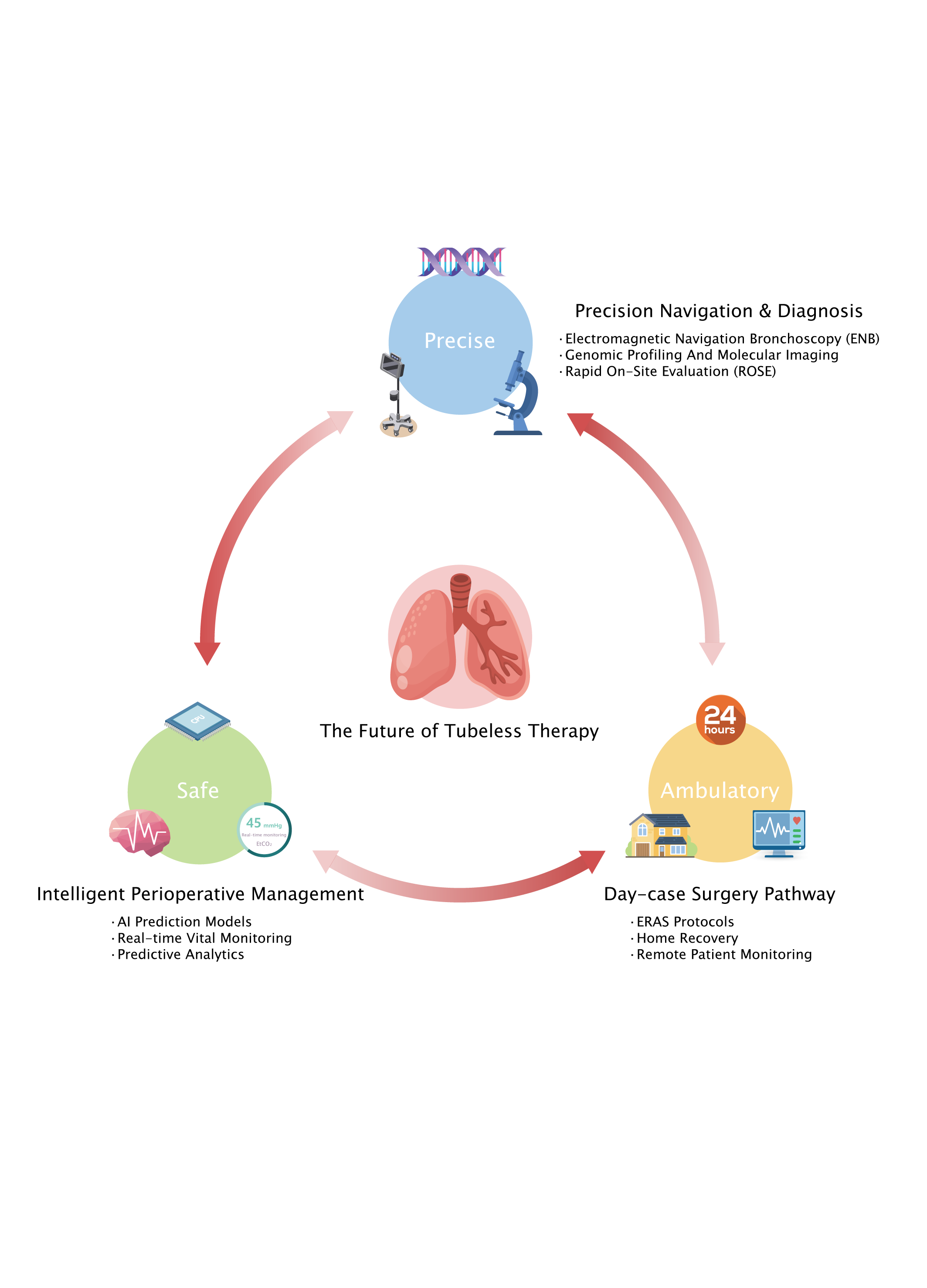


**Figure 4. The integrated innovation framework for the future evolution of Tubeless technology.**

This conceptual map illustrates three synergistic innovation drivers poised to advance Tubeless VATS: 1) Precision Navigation & Diagnosis, enhancing preoperative planning and targeting through tools like electromagnetic navigation bronchoscopy (ENB) and molecular imaging; 2) Intelligent Perioperative Management, utilizing artificial intelligence (AI) and real-time vital monitoring for real-time risk prediction; 3) Streamlined Day-Case Surgery Pathways, optimizing patient workflows through enhanced recovery after surgery (ERAS) protocols and remote monitoring. The convergence of these interdisciplinary fields is driving the evolution of Tubeless therapy toward a future paradigm that is more precise, safe, painless, and ultimately suitable for ambulatory settings.

The proposed directions are research priorities and conceptual frameworks, not established clinical standards.
