# Supplementary figures and images for: Research advances of tubeless thoracic surgery for pulmonary nodules: current status and future challenges
Source: Front Surg. 2026 May 25;13:1834893. doi: 10.3389/fsurg.2026.1834893 (PMC13243431; doi:10.3389/fsurg.2026.1834893)

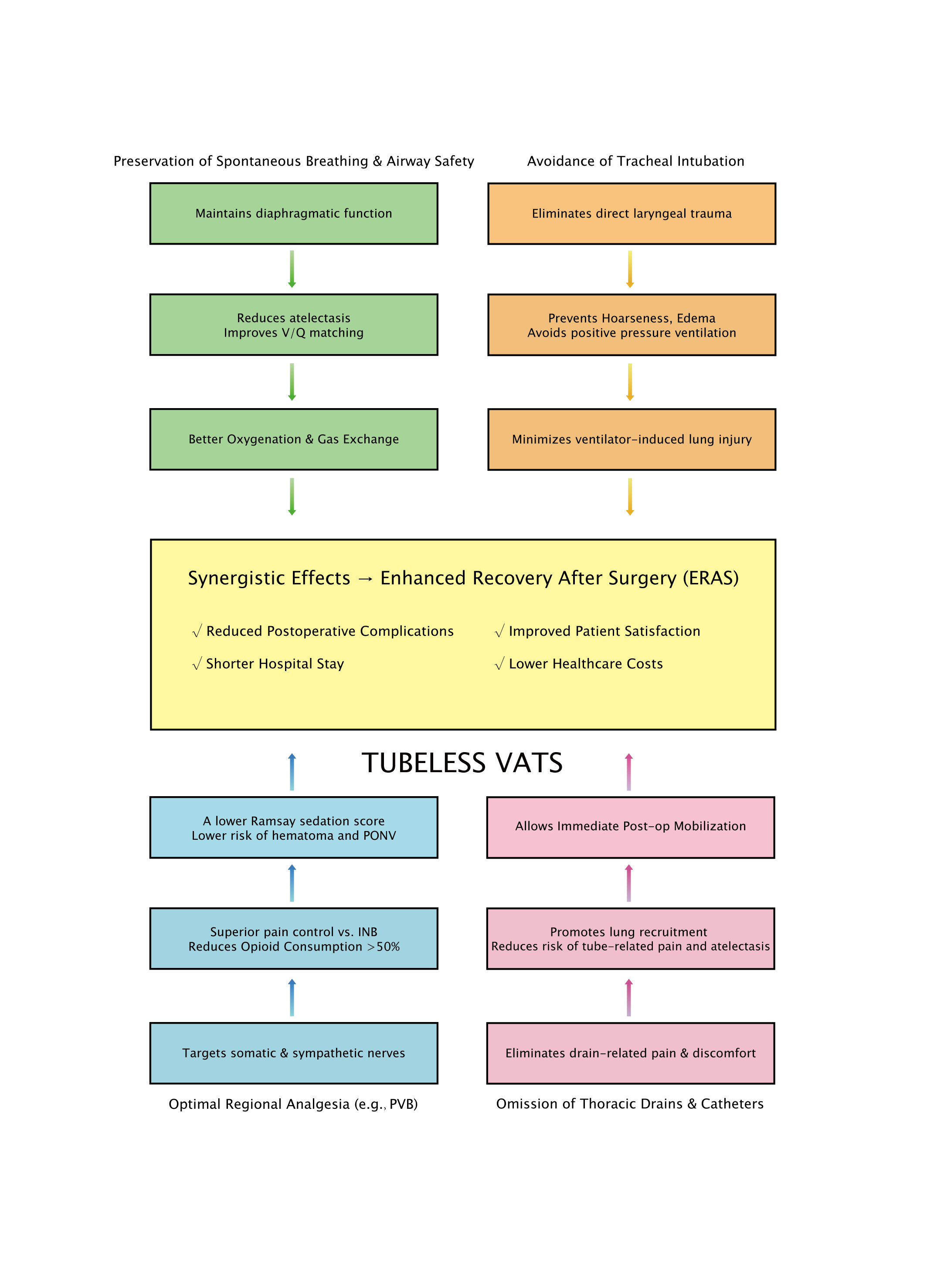

Supplement: Supplementary file 1 [file Datasheet1.zip › Supplementary material presentation/Fig.1.tif]

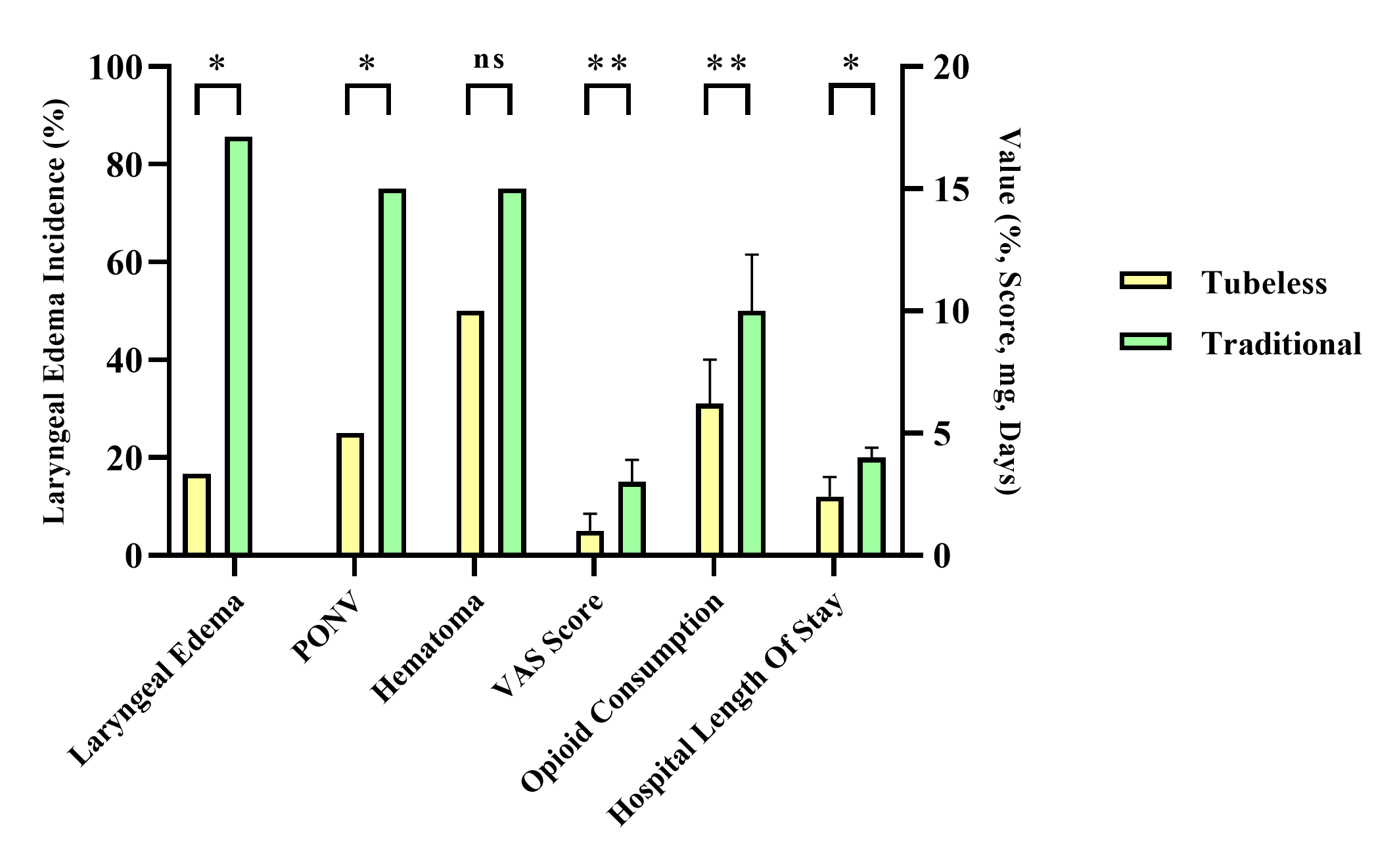

Supplement: Supplementary file 1 [file Datasheet1.zip › Supplementary material presentation/Fig.2.tif]

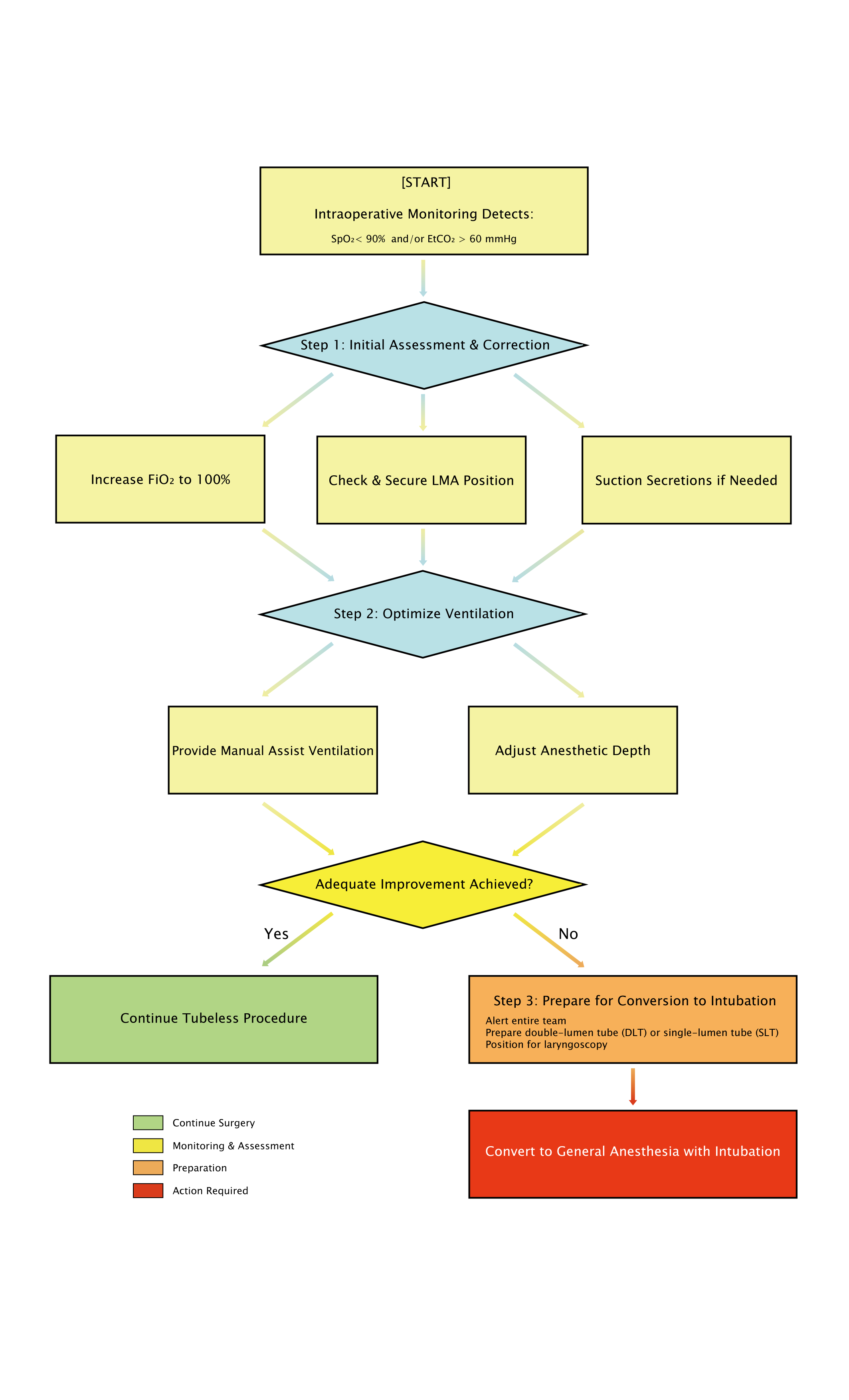

Supplement: Supplementary file 1 [file Datasheet1.zip › Supplementary material presentation/Fig.3.tif]

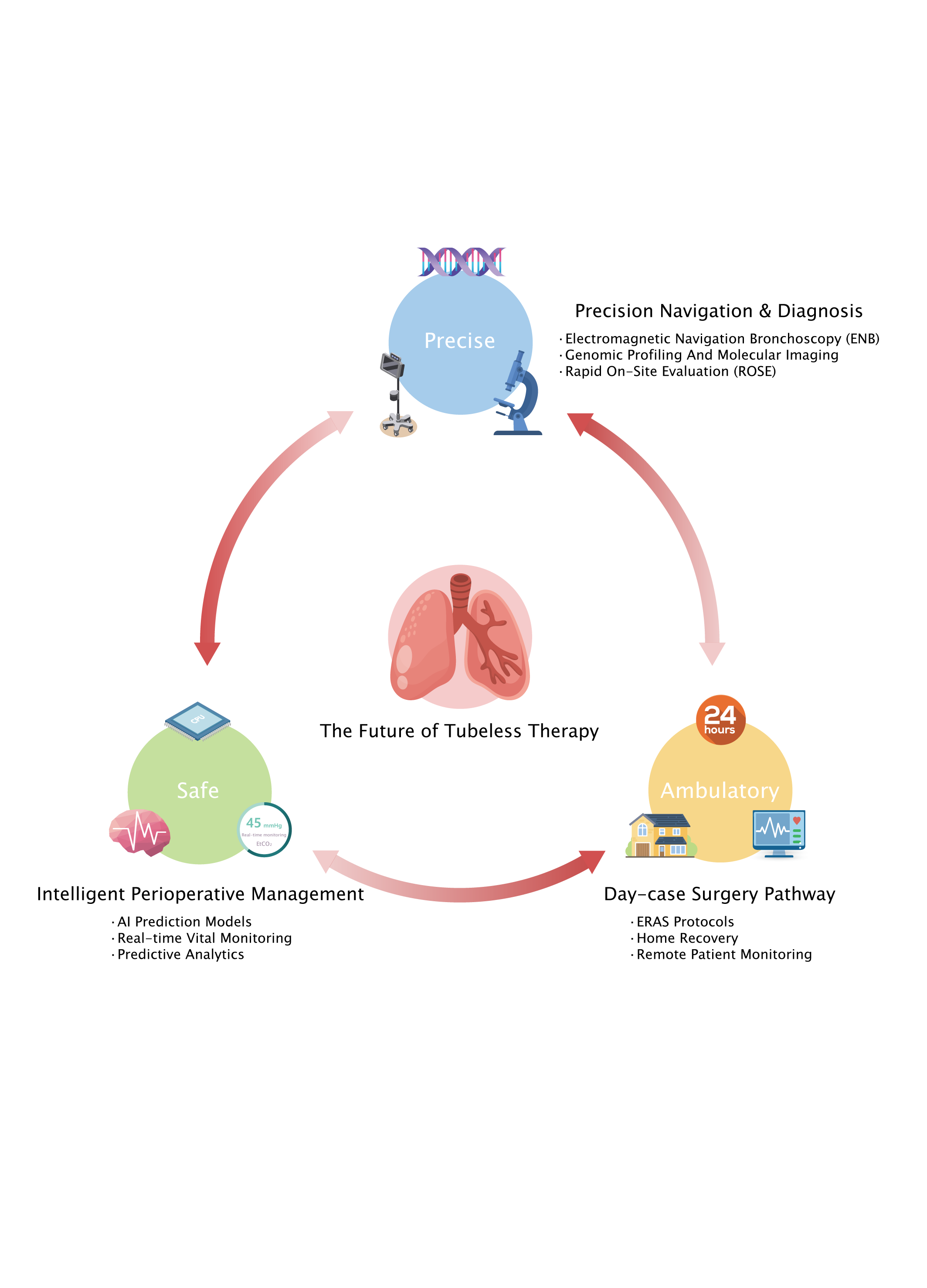

Supplement: Supplementary file 1 [file Datasheet1.zip › Supplementary material presentation/Fig.4.tif]
